# Nutrient Removal by Grain in Modern Soybean Varieties

**DOI:** 10.3389/fpls.2021.615019

**Published:** 2021-06-21

**Authors:** Michel Esper Neto, Lorena Moreira Lara, Silas Maciel de Oliveira, Rayssa Fernanda dos Santos, Alessandro Lucca Braccini, Tadeu Takeyoshi Inoue, Marcelo Augusto Batista

**Affiliations:** ^1^Departamento de Agronomia, Universidade Estadual de Maringá, Maringá, Brazil; ^2^Universidade Federal de Viçosa, Viçosa, Brazil

**Keywords:** cultivars, nitrogen, phosphorus, calcium, magnesium, nutrient concentration, sulfur

## Abstract

Knowing the nutrient removal by soybean grain harvest in different varieties, locations, and over time is essential to correctly adjust agronomic recommendations, update farmers’ practices, and increase nutrient use efficiency. A field-based research trial was carried out to assess macronutrients [nitrogen (N), phosphorus (P), potassium (K), magnesium (Mg), calcium (Ca), and sulfur (S)] removed in grain by modern soybean varieties from southern Brazil introduced between 2007 and 2016. We examined changes between our set of modern varieties and a dataset of historical values encompassing a wide range of varieties introduced before 2007. Moreover, we undertook a synthesis analysis using scientific literature published after 2007 to investigate nutrient removal by grain among modern Brazilian soybeans and a dataset that included field trials from Argentina, United States, and India. There were no yield gains across the years for modern soybean varieties introduced among 2007 and 2016 in Brazil, although the grain N and Mg concentrations decreased. Modern Brazilian soybeans increased nutrient removal compared with that by soybeans historically planted in Brazil, with 11.1, 26.9, 45.0, and 31.6% more N, P, K, and Mg removed, respectively. Our results indicated that soybean growing in Brazil removed 4.3% less N relative to the values reported in the literature dataset, whereas K removal was 21.4% greater. A significant difference was also recorded for high-yield soybean varieties, and Brazilian varieties removed 11.8% less N and 8.6% more K than varieties in the literature dataset. No differences were found among locations for P removal, averaging 4.9 kg Mg^–1^ grain. In conclusion, this study indicates that the amounts of nutrients removed by modern soybean varieties were greater relative to the historical values recorded in Brazil, excluding Ca and S. Nonetheless, in the middle to long term (10 years), a significant impact of plant breeding on grain nutrient concentration was recorded only for N and Mg. The difference in nutrient removal patterns between Brazil and other countries indicates an integrated effect of management, genotype, and environment on nutrient removal. These findings provide guidance for optimal nutrient management and specific information for plant breeding programs to understand nutrient variability.

## Introduction

Soybean [*Glycine max* (L.) Merrill] is a major leguminous crop grown throughout the world. Processed soybean grain is the world’s largest source of protein for animals, and beans’ abundant stored lipids are also widely used as oilseed (Gaonkar and Rosentrater, 2019) and as a biofuel crop (Kim and Moschini, 2018). There is great potential for soybean to continue to serve as one of the most important crops for providing protein and vegetable oil. Overall, soybean management practices are well known, and their ability to fix atmospheric nitrogen (N) eliminates or reduces the additional cost of N fertilization. Currently, the total global production of soybeans accounts for 339 million metric tons (FAO, 2020). However, although soybean shows a wide range of geographical adaptation, approximately 90% of the crop is produced by only five countries (USDA, 2020). Brazil and the United States are the world’s leading soybean producers, followed by Argentina, China, and India.

Current soybean production has been achieved through a steady yield rise (9–40 kg ha^–1^ year^–1^) over the past 4 decades (FAO, 2020). This increase is linked to the application of optimal management practices and genetic progress in the form of high-yield varieties (Lazzarotto and Hirakuri, 2009; Kahlon et al., 2011). Nonetheless, origin and prolonged periods of breeding across distinct environmental conditions may have effects on other agronomic traits. Accurate estimates in the literature have reported changes in the physiological, morphological, and nutritional traits of soybean varieties (Kuang et al., 2005; Jin et al., 2010; Bender et al., 2015). When investigating physiological differences within a group of historical varieties, Morrison et al. (1999) found that photosynthesis and stomatal conductance varied among varieties of different origins. Todeschini et al. (2019) reported that modern Brazilian varieties had higher harvest indices, biological yields, and reproductive nodes per m^–2^ than older varieties. In the United States, Ustun et al. (2001) reported striking findings in terms of oil and protein content, with considerable differences between indigenous and exogenous genotype sources.

A look at the literature shows that soybean studies on grain composition have focused on single nutrients: the majority of studies have focused on N (Divito et al., 2016; La Menza et al., 2017) and, in some instances, phosphorus (P), or potassium (K; Pettigrew, 2008). Others have also studied how specific issues such as drought stress (Dornbos and Mullen, 1992; Singh et al., 2014) during seed filling and other effects of management practices (Houx et al., 2014; Farmaha et al., 2016; Maciel de Oliveira et al., 2019) affect grain composition. Most studies dealing with genotype associations with yield and grain composition have approached differences across the years (Cregan and Yaklich, 1986; Fabre and Planchon, 2000; Balboa et al., 2018). Nonetheless, few studies have reported the effects of soybean growth under different environments on nutritional grain composition (Moreira and Moraes, 2016), especially phenotypic differences among major soybean producers (La Menza et al., 2017), such as seeds per plant, photosynthetic rate, and plant height (Jin et al., 2010; Bender et al., 2015; Todeschini et al., 2019).

Nutrients removed by harvest are an important part of the sustainability and profit mechanisms in grain production systems. An accurate and current assessment of nutrient removal allows direct measurement of the nutrient budget, helping to sustain soil fertility levels and adjust the dose and type of fertilizer applied (Laberge et al., 2009; Tiecher et al., 2017). In soybean, the use of modern high-yield varieties means that greater quantities of nutrients are removed by harvest, and nutrient removal may exceed annual replacement if replacement estimates are based on old varieties used in general crop recommendations. Several studies have reported that in the long term, underestimated nutrient replacement affects agricultural cropping systems and results in yield declines (Drechsel et al., 2001; Yu et al., 2009; Houx et al., 2014), especially when high yields are observed (Hansel et al., 2017). Moreover, the removal of base cations by accelerated leaching and grain harvesting, particularly calcium (Ca) and magnesium (Mg), are commonly regarded as the cause of a decline in soil base-cation status that leads to other problems, such as acidification, high available aluminum, and disturbances in the carbon cycle (Randall et al., 2006; Godsey et al., 2007).

A soybean yield gap of considerable magnitude is correlated with suboptimal management practices (Bhatia et al., 2008; Sentelhas et al., 2015), and adequate plant nutrition is an important step in closing this gap. Furthermore, an approach to understanding the relation between yield and nutrient removal characteristics in a wide range of nutrients and genotypes may be useful for developing new soybean varieties. Taken together, knowledge on nutrient removal by modern soybean varieties cultivated in Brazil remains limited, and information about differences in grain nutrient removal by major soybean producers throughout the world is lacking.

To the best of our understanding, there are few studies about the current nutritional status of widely planted soybean varieties in Brazil. In addition, even more scarce are studies comparing soybean nutritional trends with intrinsic historical values from Brazil and global values from literature. Thus, the aim of this work was to evaluate the amount of nutrients removed by soybean at harvest using the most cultivated soybean varieties in southern Brazil. Moreover, we performed a synthesis analysis to compare our findings with reports existing in the current literature from different locations that focus on modern soybean varieties (introduced after 2007).

## Materials and Methods

### Field Trial

We used a randomized complete block design in the field with four replications and a set of 12 modern soybean varieties used in southern Brazil. The soybean varieties were grown in the 2017/2018 crop season at four different locations in the State of Paraná: Floresta (23°35′23″ S and 52°04′21″ W), Maringá (23°02′21″ S and 52°04′56″ W), Cambé (23°15′46″ S and 51°14′62″ W), and Apucarana (23°33′39″ S and 51°22′85″ W). The plots consisted of six rows that were 5 m long and had 0.45-m spacing and sown at a rate of 266,000 seeds ha^–1^.

To assess nutrient removal by soybean at harvest, we used 12 well-planted varieties in southern Brazil. The soybean varieties studied here were introduced between 2007 and 2016. The term “modern” was used following grouping adopted in a previous study (Balboa et al., 2018), and morphologic differences were found by Todeschini et al. (2019). Further details are available in Table 1.

**TABLE 1:** Soybean varieties studied, maturation groups, type of growth, height, thousand grain mass, and introduction year.

**Variety**	**Maturation group^†^**	**Type of growth**	**Height (cm)**	**Thousand grain mass (g)**	**Introduction year**
BMX POTÊNCIA RR	6.7	Indeterminate	92	168	2007
NA 5909 RG	6.2	Indeterminate	85	157	2008
NS 4823 RR	4.8	Indeterminate	90–97	180	2008
SYN 1163 RR	6.3	Indeterminate	116	148	2011
M 6410 IPRO	6.4	Indeterminate	86	145	2011
BRS 388 RR	6.4	Indeterminate	92–113	145	2013
TMG 7062 IPRO	6.2	Semi-determinate	110	200	2013
M 5947 IPRO	5.9	Indeterminate	91	170	2013
BRS 1010 IPRO	6.1	Indeterminate	90–120	170	2014
SYN 1561 IPRO	6.1	Indeterminate	90–97	164	2015
63I64 IPRO GARRA	6.3	Indeterminate	117	189	2015
TMG 7063 IPRO	6.3	Indeterminate	90–100	191	2016

*^†^Maturity classification according to Alliprandini et al. (2009).*

The soils of the experimental areas were classified as typical Oxisols except for Maringá, which was classified as a typical Ultisol (USDA Soil Taxonomy). The climate was classified as subtropical humid mesothermal Cfa (Alvares et al., 2014), characterized by a predominance of warm summers, a low frequency of severe frosts, and rainfall concentrations in the summer period. The rainfall, temperature, planting, and harvest dates at the different sites are indicated in Table 2.

**TABLE 2:** Rainfall and maximum and minimum temperatures at the four study sites during the study period.

**Climate characteristics**	**Oct**	**Nov**	**Dec**	**Jan**	**Feb**
	**Apucarana**				
Monthly rain (mm)	274.9	172.0	274.4	148.9	78.4
Mean max. temp. (°C)	26.1	26.7	27.0	26.7	28.1
Mean min. temp. (°C)	16.0	16.7	18.3	19.4	17.2
Monthly average temp. (°C)	21.1	21.7	22.7	23.1	22.7
	**Cambé**				
Monthly rain (mm)	261.3	150.3	232.3	154.3	110.1
Mean max. temp. (°C)	28.7	28.9	28.8	28.6	29.1
Mean min. temp. (°C)	17.5	18.3	18.3	19.5	19.2
Monthly average temp (°C)	23.1	23.6	23.6	24.0	24.2
	**Maringá**				
Monthly rain (mm)	311.9	132.9	210.1	243.9	149.3
Mean max. temp (°C)	27.1	29.1	29.1	28.1	29.4
Mean min. temp. (°C)	19.1	18.8	18.9	18.9	17.8
Monthly average temp (°C)	23.1	24.0	24.0	23.5	23.6
	**Floresta**				
Monthly rain (mm)	282.3	120.4	250.3	195.5	141.3
Mean max. temp. (°C)	29.9	28.3	29.5	29.8	28.9
Mean min. temp. (°C)	19.2	19.2	19.3	18.9	19.1
Monthly average temp (°C)	24.6	23.8	24.4	24.4	24.0

Before sowing, seeds were inoculated with *Bradyrhizobium japonicum* at a concentration of 5.0 × 10^9^ viable cells per mL g^–1^ and a seed rate of 2 mL kg^–1^. All the experimental areas were fertilized based on soil fertility characteristics (Supplementary Table 1) and general guidelines for crop recommendations. Cobalt and molybdenum were added at levels of 2 and 12 g ha^–1^, respectively. A total of 90 kg ha^–1^ P_2_O_5_ and 80 kg ha^–1^ K_2_O were applied at the Floresta and Apucarana sites. In Cambé, we applied 70 kg ha^–1^ of P_2_O_5_ and K_2_O, and in Maringá, the field received 85 kg ha^–1^ of P_2_O_5_ and K_2_O. All soil fertilization was applied with the seeds at planting. Other cultivation practices, including the control of pests, diseases, and weeds, were carried out according to the Embrapa (2014).

Four central rows of soybean were manually harvested. The border effect was considered, and plants were harvested 0.75 m away from the top and bottom of the plots. The grain samples were oven-dried at 105°C to determine the grain moisture content and standardized to a dry basis (0% moisture) to obtain the yield results. Grain nutrient concentration and nutrient content were also expressed on a dry basis (Ciampitti and Vyn, 2011; Tamagno et al., 2017).

### Nutrient Measurements

Soybean grain samples were individually ground to a powder in a Wiley mill with a stainless-steel blade and passed through a 40-mesh sieve. A total of 1,000 mg of milled grain was used to determine the exported nutrients. The N concentration was determined by means of complete digestion in concentrated H_2_SO_4_ and subsequent distillation using the micro-Kjeldahl method. To obtain the total nutrient content for P, sulfur (S), calcium (Ca), Mg, and K of the soybean grains, digestion by nitric-perchloric (4:1) solution was performed. Total S was determined by applying the turbidimetry method with barium sulfate. Ca and Mg concentrations were determined using an Agilent^®^ Microwave Plasma Atomic Emission Spectrometer (MP-AES). Total K and P were determined by using a flame photometer and metavanadate colorimetry, respectively. All the analyses were performed according to Malavolta et al. (1997).

### Literature Review

To examine the validity of our findings, we compared our data with the literature. We compiled soybean studies established from 2007 to 2019 from field trials dealing with management practices, genotypes, and environmental effects. The year 2007 was chosen as the starting point considering that the soybean varieties in our study were introduced on dates ranging from 2007 to 2016. All the articles that reported studies of grain yield, grain moisture, and N, P, and K removal by grain harvest were included. The entire resultant database consisted of 313 plot-specific data from 16 datasets (Table 3). The number of observations was different among nutrient evaluations, as not all nutrients were measured in all the studies.

**TABLE 3:** General information related to the literature dataset.

**Authors**	**Locations**	**Year of publication**	***n***	**Variation source**
Chandel and Pardeshi, 2011	India	2011	8	Fertilization
Meena and Biswas, 2013	India	2013	8	Fertilization
Patel et al., 2015	India	2015	12	Fertilization
Tamagno et al., 2017	Argentina	2017	68	Fertilization
Tamagno et al., 2017	United States	2017	99	Fertilization and plant density
Balboa et al., 2018	United States	2018	27	Management intensity
Age et al., 2019	India	2019	8	Fertilization
Cordova et al., 2019	United States	2019	8	N fixation
Goswami et al., 2019	India	2019	5	Fertilization
La Menza et al., 2019	Argentina and United States	2019	26	Fertilization
Ortez et al., 2019	United States	2019	2	Fertilization
Lenka et al., 2019	India	2019	10	CO_2_ level and fertilization
Virk et al., 2020	India	2019	8	Fertilization
Yaduwanshi et al., 2019	India	2019	9	Inoculation
Raj et al., 2019	India	2019	5	Fertilization
Yadav et al., 2019	India	2019	10	Fertilization

The search keywords used were “soybean,” “grain yield,” “nutrient uptake,” “nutrient removal,” “nitrogen,” “phosphorus,” and “potassium.” We searched the Web of Science Core Collection, Scopus, Springer Link, and Google Scholar citation databases. Grain yield and nutrient grain content were all adjusted to a dry basis.

Most studies in the literature reported no data dispersion in terms of the variance, standard deviation, or standard error of their averages, making it difficult to perform a meta-analysis (Viechtbauer and Cheung, 2010). Therefore, a synthesis analysis was performed using our database. Others have employed similar approaches to evaluate a database when no variance data are available (Mourtzinis et al., 2018; Assefa et al., 2019).

To describe the differences in grain nutrient removal between the modern varieties grown in our field trial and the historical values for varieties from southern Brazil introduced before 2007, a theoretical model for nutrient removal by grain harvest was estimated based on historical values for this region. A similar approach was taken by Bender et al. (2015). The historical values reported by Pauletti et al. (2017) include numerous local but seldom published studies. Linear functions were fitted to describe historical nutrient removal (kg ha^–1^) as a function of yield (Mg ha^–1^) in the current study. The grain nutrient content was calculated by multiplying the nutrient percentage by the yield on a dry basis. The nutrient values used in the historical model were 4.7, 0.45, 1.4, 0.23, 0.18, and 0.47 g 100 g^–1^ for N, P, K, Ca, Mg, and S, respectively.

### Descriptive and Statistical Analysis

Descriptive and statistical analyses were performed in SAS (SAS Institute, 2012). Figures that document models were plotted using GraphPad Prism^®^ software. The pie chart showing relationships among nutrient concentrations was built using the CORRPLOT package in R software (R Core Team, 2018).

The PROC MEAN procedure was used for descriptive statistics and to determine the minimum, maximum, mean, interquartile range, and standard deviation. Moreover, a box plot was plotted for grain nutrient contents. The study data were subjected to an analysis of basic statistical assumptions using a Box–Cox test (Box and Cox, 1964). The four experimental sites were summarized, comprising 192 observations for all the soybean varieties. Because of the considerable range in the introduction years (2007–2016) of the varieties used herein, we examined the grain nutrient concentrations across the years. Following previous studies that investigated soybean traits over time (Jin et al., 2010; Todeschini et al., 2019), the values of the agronomic traits (nutrient concentration and nutrient removal) were plotted against the year of variety release, and linear regression analysis was performed to illustrate the changes. Afterward, associations among the grain nutrient concentrations were determined by Pearson correlation analysis.

To investigate grain nutrient removal, we built linear regression models using the zero intercept for the observed data points (Balboa et al., 2018), with nutrient removal as a function of grain yield. Significance of the function models was tested at *p* < 0.05. Data from the present study and the literature were also compared. An *F* test (honest difference test; Mead et al., 1993) was performed to compare the slopes of linear functions, and a single linear function between the study and literature data was calculated when no statistically significant difference (*p* > 0.05) was found. In accordance with previous studies (Coombs et al., 2017), the percent of variance (*σ*^2^ or *R*^2^) for the yield and grain nutrient relationships among models was determined by linear regression between the residuals of the grain nutrient concentration and grain nutrient content (Sadras et al., 2016) against yield (Supplementary Figures 1, 2).

To measure the proportional change between yield and nutrient removal among the datasets, we calculated a change index for grain yield and nutrient removal. These calculations were made according to Sadras et al. (2016):

(1) $$\text{Change}\left(\%\right)=\frac{\sum \left[\left(\frac{x\,i}{y}\right)-1\right]}{n}\times 100$$

where Σ is the sum, *xi* represents a term (yield or nutrient removal) in our experimental dataset, *y* is the overall mean of the yield or nutrient removal for the dataset of interest (literature or historical), and *n* is the number of observations of the present study (*n* = 192).

Changes in nutrient removal were plotted against changes in grain yield only compared to the literature, as the amount of nutrient removal estimated for historical values was calculated using the yield of our study. In addition, to investigate the nutritional change patterns of high-yield soybean varieties (>4 Mg ha^–1^; Farmaha et al., 2016; Kaschuk et al., 2016), a change index was calculated to include all observations.

## Results

### Year of Variety’s Release and Agronomic Traits

**FIGURE 1:**
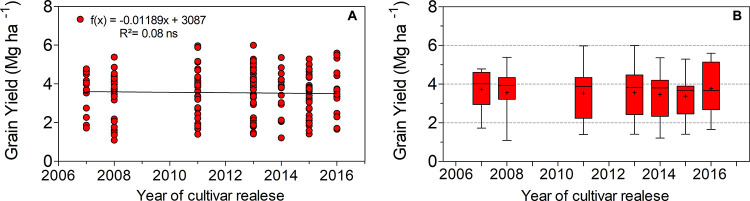
**(A)** Relationship between the years of varieties’ releases and yield, and **(B)** box plot diagrams of yield indicating the variability outside the upper and lower quartiles across years (*n* = 192).

**FIGURE 2:**
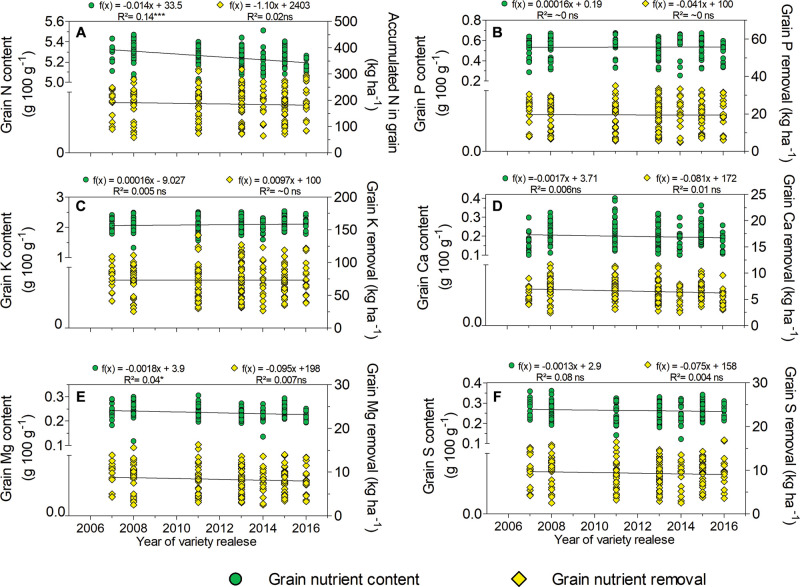
Relationship between the years of varieties’ releases and grain nutrient content of soybean or amount of nutrient removal in the current study (*n* = 192): **(A)** nitrogen, **(B)** phosphorus, **(C)** potassium, **(D)** calcium, **(E)** magnesium, and **(F)** sulfur. ns = not significant. *Significant at 5% and ***significant at < 0.001%.

A linear regression equation showed no significant trends between yield and the years of variety release (2007–2016), averaging 3.5 Mg ha^–1^ (Figure 1A), with a minimum of 1.9 Mg ha^–1^, and a maximum of 6.5 Mg ha^–1^ (Figure 1B). A slight decreasing trend was found in N and Mg grain concentrations across years of variety release (Figures 2A,E). The observed rates of decrease were 0.014 and 0.0018 g 100 g^–1^ year^–1^ of grain per year for N and Mg, respectively. Our results indicate no differences in grain concentrations of P, K, Ca, or S for the varieties introduced since 2007, averaging 0.5, 2.0, 0.2, and 2.6 g per 100 g of grain, respectively (Figures 2B–D, F). The amounts of grain nutrient removal among the nutrients were stable in the varieties introduced since 2007 (Figure 2). The average amounts of grain nutrients removed were 214, 22, 85, 8.0, 9.5, and 11 kg ha^–1^ for N, P, K Ca, Mg, and S, respectively.

### Nutrient Correlations

**FIGURE 3:**
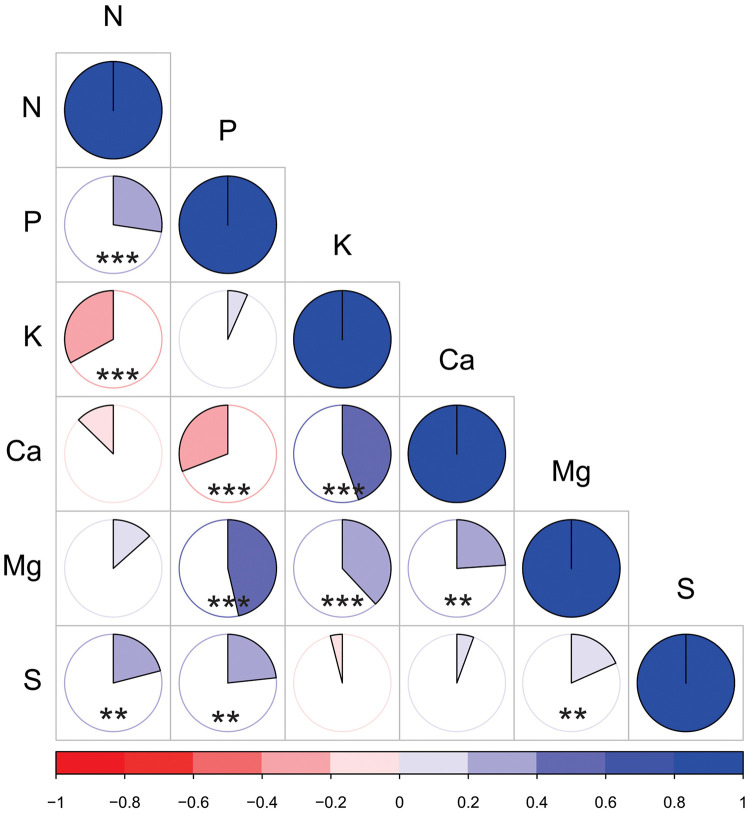
Pearson correlation coefficient *r* values among grain nutrients (g 100 g^–1^). Positive correlations are displayed in blue, and negative correlations are displayed in red. The color intensity and arc length of each slice are proportional to the correlation coefficients. **Significant at 0.01% and ***significant at < 0.001%.

Figure 3 summarizes the nutrient correlations for soybean grain (g 100 g^–1^) performed in this study. Our results indicated that N × P (*r* = 0.37^∗∗∗^), N × S (*r* = 0.21^∗∗∗^), K × Ca (*r* = 0.40^∗∗∗^), K × Mg (*r* = 0.37^∗∗∗^), Ca × Mg (*r* = 0.41^∗∗^), P × Mg, P × S, and S × Mg (*r* = 0.18^∗∗∗^) had positive correlations, whereas N × K (*r* = –0.37^∗∗∗^) and P × Ca (*r* = –0.41^∗∗∗^) were negatively correlated. Correlations among P × K, N × Ca, N × Mg, S × K, and S × Ca were not significantly different, indicating zero interaction.

### Changes in Nutrient Removal Among Datasets

**FIGURE 4:**
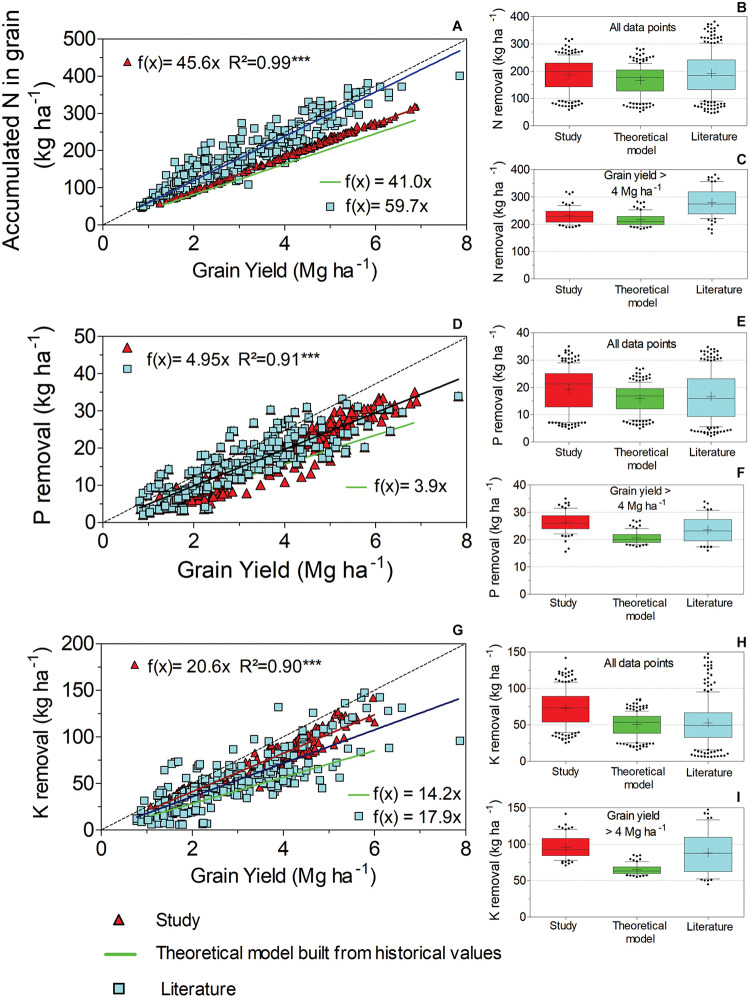
Linear functions to describe N, P, and K removal relative to grain yield **(A, D, G**, respectively). Differences among the datasets were tested through an *F* test (*p* < 0.05). When no differences were found between the study and literature, a single model was pooled (indicated with a black line). Box plots **(B, E, H)** display the data distribution of the overall yield for N, P, and K removal by harvest. Box plots **(C, F, I)** display the distribution of yield data above 4 Mg ha^–1^ for N, P, and K removal by harvest. ***Significant at < 1%.

The relationship between accumulated N in grain and yield showed significant differences (*p* < 0.05) among all the datasets (Figure 4A). A greater slope was found in the literature dataset (59.7 kg N Mg^–1^ grain), which included studies from Argentina, India, and the United States. The slope between accumulated N in grain and yield for our study (45.6 kg N Mg^–1^ grain) was greater than that of the model built from historical values, which included regional varieties introduced before 2007 (41 kg N Mg^–1^ grain). The box-and-whisker plots of the nutrients removed were created to display the distribution for each dataset, which also included a separate analysis for data points classified as high yield (>4 Mg ha^–1^). Details of the descriptive analysis are available in Supplementary Table 2. On average, accumulated N for all the yield data in our study, historical values, and the literature was 186, 167, and 197 kg ha^–1^, respectively (Figure 4B). In high-yield soybean varieties, the amount of N removed always remained above 200 kg N ha^–1^, averaging 230, 217, and 279 kg ha^–1^ for this study, the historical dataset, and the literature dataset, respectively (Figure 4C).

Our study and literature datasets showed similar relationships between P removal and yield (*p* > 0.05), resulting in P removal of 4.95 kg Mg^–1^ grain (Figure 4D). However, the mean P removal in both datasets was greater than that in the model created using historical values (3.9 kg P Mg^–1^ grain, *p* < 0.05). Considering all the yield data, the total P removed in the study, historical, and literature datasets was 19, 15.9, and 16.4 kg ha^–1^, respectively (Figure 4E). For the high-yield soybean varieties, we observed 26 kg ha^–1^ of P removal in our study, whereas the nutrient removal in the historical and literature datasets was 21 and 24 kg ha^–1^, respectively (Figure 4F).

When we analyzed the relationship between K removal and yield, we found significant differences (*p* < 0.05) among the three datasets (Figure 4G). A greater amount of K removal by harvest was recorded for soybean growing in our field study (20.6 kg K Mg^–1^ grain), followed by the literature dataset (17.9 kg K Mg^–1^ grain) and the model predicted using historical values (14.2 kg K Mg^–1^ grain). Overall, the total K removed from the study, historical, and literature datasets was 73, 50, and 59 kg ha^–1^, respectively (Figure 4H). The high-yield soybean varieties in the current study accounted for a K removal of 96 kg ha^–1^ (Figure 4I). For the model built from historical values and K removal observed from the literature dataset for the high-yield soybean varieties, the total nutrient removal was 65 and 88 kg K ha^–1^.

**FIGURE 5:**
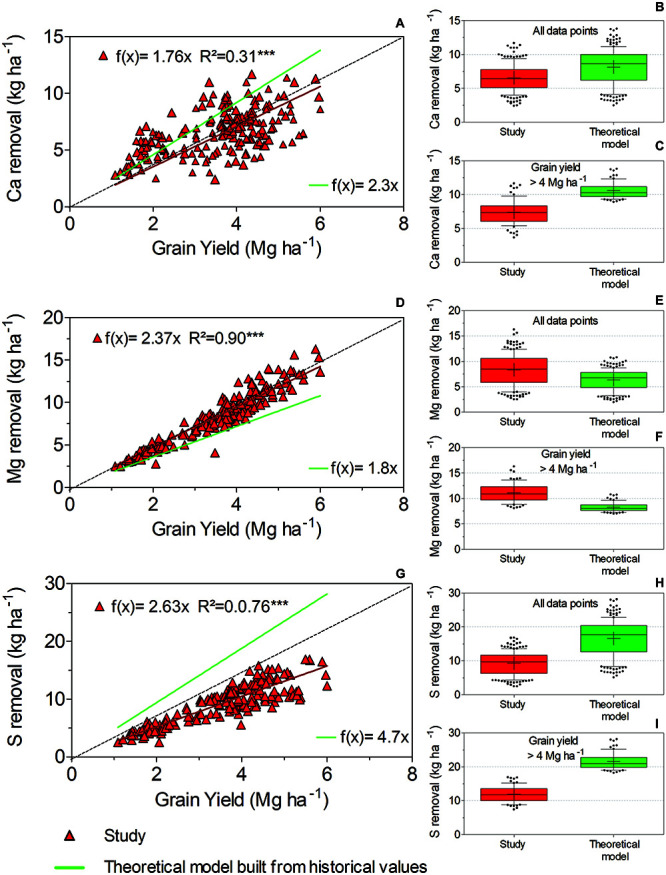
Linear functions to describe **(A)** Ca, **(D)** Mg, and **(G)** S removal relative to grain yield. Differences among the datasets were tested using an *F* test (*p* < 0.05). The box plots display the data distribution of the overall yield for **(B)** Ca, **(E)** Mg, and **(H)** S removal by harvest. Box plots **(C)**, **(F)**, and **(I)** display the distribution of yield data above 4 Mg ha^–1^ for **(C)** Ca, **(F)** Mg, and **(I)** S removal by harvest.

Figure 5A shows the relationship of Ca removal with yield. The slope of the model built from the current study data, reaching 1.76 kg Ca Mg^–1^ grain, was significantly lower (*p* < 0.05) than that derived from historical values (2.3 kg Ca Mg^–1^ grain). The slope of Mg removal to yield was greater for this study than for the estimated model derived from the historical dataset (*p* < 0.05). The slopes were 2.37 and 1.80 kg Mg^–1^ grain for the data from this study and the historical dataset, respectively (Figure 5D). Finally, the relationship between S removal and yield also showed significant differences (*p* < 0.05) between datasets. Our study showed a lower slope (2.63 kg S Mg^–1^ grain) relative to the slope of the model built from historical values (4.7 kg S Mg^–1^ grain). The distributions of Ca, Mg, and S removal for all the yield data (Figures 5B,E,H) and high-yield soybean varieties (Figures 5C,F,I) were calculated. Upon analyzing all the yield data of the study, the average values of Ca, Mg, and S removal were 6.6, 8.4, and 9.3 kg ha^–1^, respectively. The model built from historical values resulted in average removal values of 8.2 kg Ca ha^–1^, 6.4 kg Mg ha^–1^, and 16.7 kg S ha^–1^. For the high-yield soybean varieties in our study, the nutrient removal values were as follows: 7.4 kg Ca ha^–1^, 11 kg Mg ha^–1^, and 12 kg S ha^–1^. The model built from historical values indicated a total nutrient removal of 10.6 kg Ca ha^–1^, 8.3 kg Mg ha^–1^, and 21.6 kg S ha^–1^.

Residuals of the adjusted functions describing nutrient removal were also calculated and plotted against yield (Supplementary Figures 1A–C, 2A–C). All the residuals indicated random dispersion and good fit for our linear functions (Figures 4, 5). Moreover, the residuals between nutrient removal and yield plotted against nutrient concentration show that a significant proportion of the variation found in nutrient removal accounted for changes in grain nutrient concentration (Supplementary Figures 1D, 2D).

**FIGURE 6:**
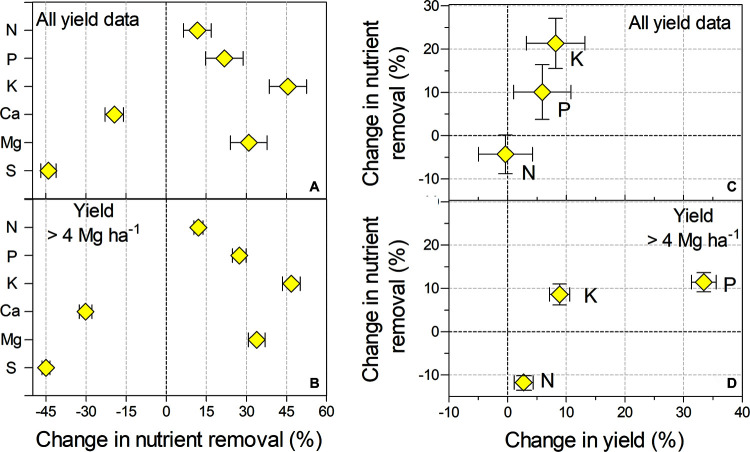
Relative change in nutrient removal as a function of **(A)** relative yield and **(B)** relatively high yield for the current study and model built from historical values. **(C)** Relative change in nutrient removal (N, P, and K) as a function of relative yield and **(D)** relatively high yield for the current study and literature data. The vertical and horizontal bars indicate the standard error for the *x* and *y* variables, respectively.

The proportional change between yield and nutrient removal between our study and the model built from historical values is presented in Figure 6 for all the yield data and for those soybean strains that yielded more than 4 Mg ha^–1^. Considering all the yield data, we observed positive change values relative to the historical model for N, P, K, and Mg, indicating greater nutrient removal by the strains used in our study (Figure 6A). On the other hand, we observed negative values of change for S and Ca. Similar trends were observed between our study and historical values for the high-yield soybean varieties (Figure 6B).

Negative values at the *x* axis and *y* axis indicate that the yield and accumulated N in grain reported in the literature were greater than those in this study when including all the yield data (Figure 6C), but the relative variation in yield (–0.4%) was small compared with the variation in accumulated N in grain (–4.3%). We recorded positive values for P and K for both yield and nutrient removal. Nonetheless, the relative variation in yield was also small relative to P (10.1%) and K (21.4%) removal. The change in nutrient removal as a function of high-yield soybean varieties is presented in Figure 6D. Relative variations were quite ample for accumulated N in grain (–11.8%) but not for yield (2.7%). On the other hand, we found a relative variation of 11.7% for P removal and 33% for yield. The relative variations in K removal (8.6%) and yield (8.9%) were very close to 1:1 in the high-yield soybean varieties.

## Discussion

The present study showed that in the medium to long term, yield trends were not associated with the years of soybean variety release in southern Brazil (Figure 1). The yield gain across years is a well-documented effect of plant breeding on soybean (Jin et al., 2010; Todeschini et al., 2019), although the short period measured and mean yield reached in this study likely led to a yield plateau. The mean yield (dry weight basis) recorded in our study (3.5 Mg ha^–1^) was ∼25% above the average rain-fed on-farm soybean yields in Brazil (2.6 Mg ha^–1^; USDA, 2020), and yield plateaus are more frequently observed in moderate- to high-yield environments (Duvick and Cassman, 1999; Egli, 2008).

The decrease in N grain concentration across the years of variety release found herein (0.014 g 100 g^–1^ year^–1^) is consistent with the long-term studies by Ustun et al. (2001) and Wilson et al. (2014), who observed rates of decrease ranging between 0.012 and 0.024 g 100 g^–1^ year^–1^. Concerning Mg grain concentration, although previous studies also found a wide range of concentrations (0.17–0.34 g 100 g^–1^) due to varietal differences (Raboy et al., 1984; Moraghan et al., 2006), no correlation linking Mg grain concentration with variety release years was observed in our study (Figure 2). The trend observed for N and Mg grain concentrations over a short time suggests a greater impact of plant breeding on both nutrients for soybean varieties growing in southern Brazil. Moreover, trends of N and Mg grain concentrations may be less impacted by yield for Brazilian soybean varieties as changes were observed in the absence of grain yield. Certainly, changes in the concentrations of N and Mg in grains result from interactions among the environment, genotype, and management approaches. Changes in N and Mg grain concentrations due to climate and soil fertility are unlikely as the cultivars were cultivated under the same conditions. From a genetic point of view, a possible cause of the N and Mg decrease is related to the selection of genotypes with early cycles. Studying morphological changes of soybean cultivars over the years in Brazil, Todeschini et al. (2019) reported a reduction in the number of days for maturation of 0.29 days year^–1^. A short growth cycle can reduce the time for accumulation and redistribution, affecting grain nutrient concentrations.

The modern soybean varieties studied herein removed more N, P, K, and Mg than the historical varieties planted in Brazil; on the other hand, changes in S and Ca removal were not significant. The specific mobility of each nutrient is influenced by the functions that nutrients play in plant metabolism and determines their mobility or redistribution within the plant. The macronutrients N, P, and K have high mobility and are easily redistributed inside plants (Bernardi et al., 2000), as well as grains. On the other hand, the mobility of S and Ca within a plant can be considered low, which means that these nutrients are less mobile and less likely to be distributed within plants and grains (Mascarenhas et al., 2013).

A large fraction of available plant nutrients are allocated into grains, and investigating nutrient correlations provides an opportunity for optimizing nutrient synergism and plant nutrient utilization. As mentioned previously, the N grain content decreased across the years, and the consistent positive correlation among N, P, and S (Figure 3) found in our study suggests that pairwise combinations of N, P, and S may avoid further N depletion in soybean grains. Previous studies have found enhanced growth of leguminous species (DiTommaso and Aarssen, 1989; Kuang et al., 2005), as well as biological N fixation (Sexton et al., 1998), when both P and S fertilization were performed in the field, indicating P and S supply as hotspots for crop growth and N nutrition. In addition to rhizobia serving as a strong sink for P (Almeida et al., 2000), nutrients play an important role in the metabolic processes of N-fixing symbiosis and the synthesis of nucleic acids (O’Hara, 2001). Moreover, an adequate S supply enhances the total number of root nodules (Scherer et al., 2006) and their nitrogenase activity (Jeong and Jang, 2006). A positive correlation among K, Ca, and Mg was also observed in our study. These cationic nutrients are taken up by root plants, but different patterns in the accumulation of nutrients were observed in aboveground soybean tissues such as leaves and steam. Overall, the ratio of K, Ca, and Mg is affected by soybean variety, growth stage (Jiménez et al., 1996), and available nutrients for uptake in soil solution (Loide, 2004). In our study, we found a positive and steady correlation among K, Ca, and Mg grain contents, indicating that changes in the grains were hardly ever observed relative to changes in other plant organs and that grains are likely less sensitive to changes in soil solution.

Our study portrayed significant effects on the rates of N and K removal by modern Brazilian soybean varieties relative to the effects exerted by the varieties in the literature dataset, which included field trials in Argentina, the United States, and India. We observed greater accumulated N in grain for the soybean varieties grown in Brazil. Studies dealing with modern soybean genotypes in the United States and Argentina have reported high N allocations from stover to grain (Rotundo et al., 2014; Tamagno et al., 2020), which indicates the ability of these genotypes to maintain grain N content. This may explain the differences in accumulated N in grain between genotype sources. A retrospective study testing the N content ratio between grains and whole plants [N harvest index (NHI)] in Brazilian germplasm is missing to date. However, NHI values available in the literature range from 52 to 72 for soybean growing in Brazil (Alves et al., 2003; Moreira et al., 2016; Maciel de Oliveira et al., 2019), small values compared to the NHI measured in the United States and Argentina (83–90). Moreover, accumulated N in grain is an important component of the N balance in the soil–plant system, and the differences found in our study for accumulated N in grain indicated different N demands among the sites studied herein, which is useful information for further studies intending to increase soybean yield by additional N supply (e.g., cover crop residues and N fertilization).

The rate of K removal for our set of varieties was greater than that found in the literature dataset (Figure 4G), but the overall means among the datasets were similar concerning high-yield soybean varieties (Figures 4E,F). The difference may indicate luxury uptake under low to moderate yields for Brazilian soybean genotypes, a phenomenon that occurs when the concentration of nutrients increases in a tissue without a corresponding increase in yield (Kang et al., 2015; Zhao et al., 2019). Therefore, further studies should examine the effect of the K rate on yield, as K recommendations based on nutrient removal may overestimate crop K needs for this range of yields.

No genotypic effect was observed on P removal (Figure 4D), indicating that trends in P are more stable than those of N and K for modern soybean varieties. These results corroborate the findings of Balboa et al. (2018), who investigated nutritional changes over time. They observed a stable relationship between P uptake and yield for soybean varieties introduced after 1997.

Our results, from a diverse range of modern varieties, indicate an underestimation of Mg removal relative to the theoretical model built from historical values (Figure 5G), which can lead to inappropriate Mg management. Despite the lack of hard data on yield loss from Mg deficiency, a recent study in Brazil investigating soybean response to additional Mg fertilization recorded a yield gain from 0.3 to 0.8 Mg ha^–1^ (Altarugio et al., 2017). In southern Brazil, as well as in most tropical and subtropical areas, soybean grows under highly leached soil conditions characterized by low cation exchange and soil organic matter (Yang et al., 2017). Therefore, yield loss associated with an inadequate replenishment of cations such as Mg is likely, and the updated data available here may help producers meet nutritional soybean needs. Bender et al. (2015) also compared previous reports and an observational dataset that included modern soybean varieties growing in the northern United States but found an opposite Mg removal trend, indicating that modern soybean varieties have a lower rate of Mg removal than older varieties. Apparently, a distinct effect of plant breeding on nutritional trends between Brazilian and United States soybean varieties should once again be noted.

Figure 6B summarizes the results of a comparison between our set of varieties and those in the literature dataset. Three conclusions can be drawn. First, the ratios support the hypothesis that differences were associated with soybean genotype traits instead of asymmetric yield distribution. Second, the changes in N and K removal were larger than the changes in yield; these differences likely became more pronounced as yield improved, particularly for N. Finally, our results quantify a pattern of nutrient removal, highlighting the need to account for these differences when proposing a nutritional budget between Brazilian and other sites.

The relative changes in nutrient removal between our study and the theoretical model built from historical values ranged from –45 to 45% across nutrients (Figure 6A). This wide range in rates of nutrient removal also illustrates variation between old and new Brazilian soybean varieties, and differences are especially apparent for K and Mg. In Brazil, the soybean yield gap caused by crop management is mainly reported in southern Brazil (Sentelhas et al., 2015), and nutritional restrictions are a considerable part of that gap (Van Ittersum et al., 2013).

Our study showed that the removal of mineral macronutrients is a general but important mechanism. A focus on nutrient removal patterns and their relations is likely to provide better tools for future nutrient management and help design breeding strategies to meet specific goals, such as greater protein content and food biofortification, which in the current context of hunger eradication have gained relative prominence. Nevertheless, yield is still the major target in most breeding programs worldwide. Thus, changes in nutrient exports may occur indirectly by material selection with different nutrient requirements, even though nutrient content is a focus of breeding in the short term.

## Conclusion

The set of modern soybean varieties we studied shows a change in nutrient removal trends between old and new varieties growing in southern Brazil. The introduction of new soybean varieties resulted in greater removal of N, P, K, and Mg relative to the old soybean varieties, suggesting that these nutrients can significantly influence yield, and use of field-specific management may be needed to reach nutritional requirement. Analysis of grain yield showed that in the medium to long term, yield trends were not associated with the years of soybean variety release, indicating no yield gains for modern soybean varieties introduced between 2007 and 2016 in Brazil.

From this study, it was evident that the amount of N and K removed by modern soybean varieties at harvest were affected among locations. Brazilian varieties removed less (–4.3%) N and more K (21.4%) than soybean varieties in the literature dataset, and more relevant differences were observed in high-yield soybean for N (–11.8%). No differences were found among locations for P removal. These results revealed significant variability in N and K removal due to location effect and suggest that soybean growing in Brazil can meet high-yield with less N relative to the other locations studied, such as Argentina, India, and the United States. Additional studies are needed to further elucidate how soil properties, management, genotype, and environment affect nutrient removal among different locations.

## Data Availability Statement

The original contributions presented in the study are included in the article/Supplementary Material; further inquiries can be directed to the corresponding author/s.

## Author Contributions

ME, LL, RS, and SO designed the experiments and wrote the manuscript. MB, TI, and AB edited the manuscript. ME, LL, RS, and MB performed the experiments. ME and SO analyzed the data. All authors contributed to the article and approved the submitted version.

## Conflict of Interest

The authors declare that the research was conducted in the absence of any commercial or financial relationships that could be construed as a potential conflict of interest.
