# Supplementary material for: Nutrient Removal by Grain in Modern Soybean Varieties
Source: Front Plant Sci. 2021 Jun 21;12:615019. doi: 10.3389/fpls.2021.615019 (PMC8256798; doi:10.3389/fpls.2021.615019)
Supplement: Supplementary file 1 [file Data_Sheet_1.docx]

Supplementary Table 1 Soil chemical characteristics in the experimental area before the plating. Soil test was conducted at the 0-20 cm soil depth.

| Site | pH^1^ | P^3^ | SO_4_^-2^ | K^+^ | Ca^2+^ | Mg^2+^ | H+Al | Cu^2+^ | Fe^2+^ | Zn^2+^ | Mn^2+^ |
| --- | --- | --- | --- | --- | --- | --- | --- | --- | --- | --- | --- |
|  | CaCl_2_ | --mg dm^-3^-- | | ---------cmol_c_ dm^-3^---------- | | | | --------------mg dm^-3^-------- | | | |
| Floresta | 5.0 | 17.9 | 6.1 | 0.21 | 7.2 | 1.6 | 5.9 | 16.9 | 52.3 | 15.6 | 107.5 |
| Maringá | 4.8 | 6.2 | 5.2 | 0.20 | 2.8 | 1.4 | 4.1 | 14.6 | 148.6 | 6.6 | 99.5 |
| Cambé | 5.9 | 18.2 | 4.4 | 0.98 | 9.6 | 3.9 | 3.8 | 24.6 | 95.1 | 5.5 | 120.1 |
| Apucarana | 5.5 | 15.2 | 8.2 | 0.68 | 6.4 | 1.5 | 4.6 | 20.2 | 31.5 | 4,8 | 149.2 |

^1^pH in CaCl_2_ (0,01 mol L^-1^),  ^3^P, K, Cu, Fe, Zn e Mn extracted by Mehlich-1 solution; Ca and Mg extracted by KCl 1,0 mol L^-1^; H+Al determined by SMP method.

Supplementary Table 2 Descriptive analysis for grain yield, grain nutrient removal and grain nutrient concentration, calculating minimum, maximum, mean, 25 and 75% percentile, and standard deviation (SD).

| Source of  variation | Min. | Max. | Mean | 25%  Perc. | 75%  Perc. | SD |  |  | Min. | Max. | Mean | 25%  Perc. | 75%  Perc | SD |  |  |
| --- | --- | --- | --- | --- | --- | --- | --- | --- | --- | --- | --- | --- | --- | --- | --- | --- |
|  | Grain nutrient removal  (kg ha^-1^) | | | | | |  |  | Grain nutrient concentration  (g 100 g^-1^) | | | | | |  |  |
|  | Study | | | | | | | | | | | | | |  |  |
| Nitrogen | 58 | 318 | 186 | 142 | 230 | 61 |  |  | 4.9 | 5.5 | 5.3 | 5.2 | 5.3 | 5.3 |  |  |
| Phosphorus | 4.8 | 35.0 | 19.4 | 12.9 | 25.1 | 7.9 |  |  | 0.3 | 0.7 | 0.5 | 0.5 | 0.6 | 0.1 |  |  |
| Potassium | 25 | 142 | 73 | 54 | 89 | 25 |  |  | 1.3 | 2.5 | 2.1 | 1.9 | 2.3 | 0.2 |  |  |
| Calcium | 2.4 | 11.7 | 6.6 | 5.2 | 7.8 | 2.0 |  |  | 0.07 | 0.40 | 0.20 | 0.15 | 0.24 | 0.1 |  |  |
| Magnesium | 2.5 | 16.3 | 8.3 | 5.9 | 10.6 | 3.1 |  |  | 0.12 | 0.31 | 0.23 | 0.22 | 0.25 | 0.0 |  |  |
| Sulfur | 2.5 | 16.9 | 9.3 | 6.4 | 11.7 | 3.4 |  |  | 0.12 | 0.36 | 0.26 | 0.23 | 0.30 | 0.0 |  |  |
|  | Literature | | | | | | | | | | | | | |  |  |
| Nitrogen | 46 | 401 | 191 | 46 | 401 | 78 |  |  | 3.4 | 10.7 | 6.2 | 3.4 | 10.6 | 1.1 |  |  |
| Phosphorus | 2.1 | 34.0 | 15.2 | 2.1 | 33.9 | 7.6 |  |  | 0.2 | 1.0 | 0.5 | 0.2 | 1.1 | 0.2 |  |  |
| Potassium | 6 | 148 | 53 | 33 | 67 | 31 |  |  | 0.3 | 4.7 | 1.8 | 0.3 | 4.6 | 0.7 |  |  |
|  | Theoretical model built from historical values | | | | | | | | | | | | | |  |  |
| Nitrogen | 51 | 283 | 167 | 127 | 205 | 54 |  |  | 4.9 | 5.5 | 5.3 | 5.2 | 5.3 | 1.1 |  |  |
| Phosphorus | 4.9 | 27.0 | 15.9 | 12.1 | 19.5 | 5.2 |  |  | 0.3 | 0.7 | 0.5 | 0.5 | 0.6 | 1.0 |  |  |
| Potassium | 15 | 85 | 50 | 38 | 62 | 16 |  |  | 1.3 | 2.5 | 2.1 | 1.9 | 2.3 | 2.2 |  |  |
| Calcium | 2.5 | 13.8 | 8.1 | 6.2 | 10.1 | 2.7 |  |  | 0.07 | 0.40 | 0.20 | 0.15 | 0.24 | 0.63 |  |  |
| Magnesium | 2.0 | 10.8 | 6.4 | 4.8 | 7.8 | 2.1 |  |  | 0.12 | 0.31 | 0.23 | 0.22 | 0.25 | 0.26 |  |  |
| Sulfur | 5.1 | 28.2 | 16.6 | 12.7 | 20.5 | 5.4 |  |  | 0.12 | 0.36 | 0.26 | 0.23 | 0.30 | 0.43 |  |  |
|  | Grain yield  (Mg ha^-1^) | | | | | | | | | | | | | |  |  |
| Study | 1.1 | 6.0 | 3.5 | 2.7 | 4.3 | 1.1 |  |  |  |  |  |  |  |  |  |  |
| Literature | 0.8 | 7.9 | 3.1 | 2.0 | 4.1 | 1.4 |  |  |  |  |  |  |  |  |  |  |
| Estimated | 1.1 | 6.0 | 3.5 | 2.7 | 4.3 | 1.1 |  |  |  |  |  |  |  |  |  |  |


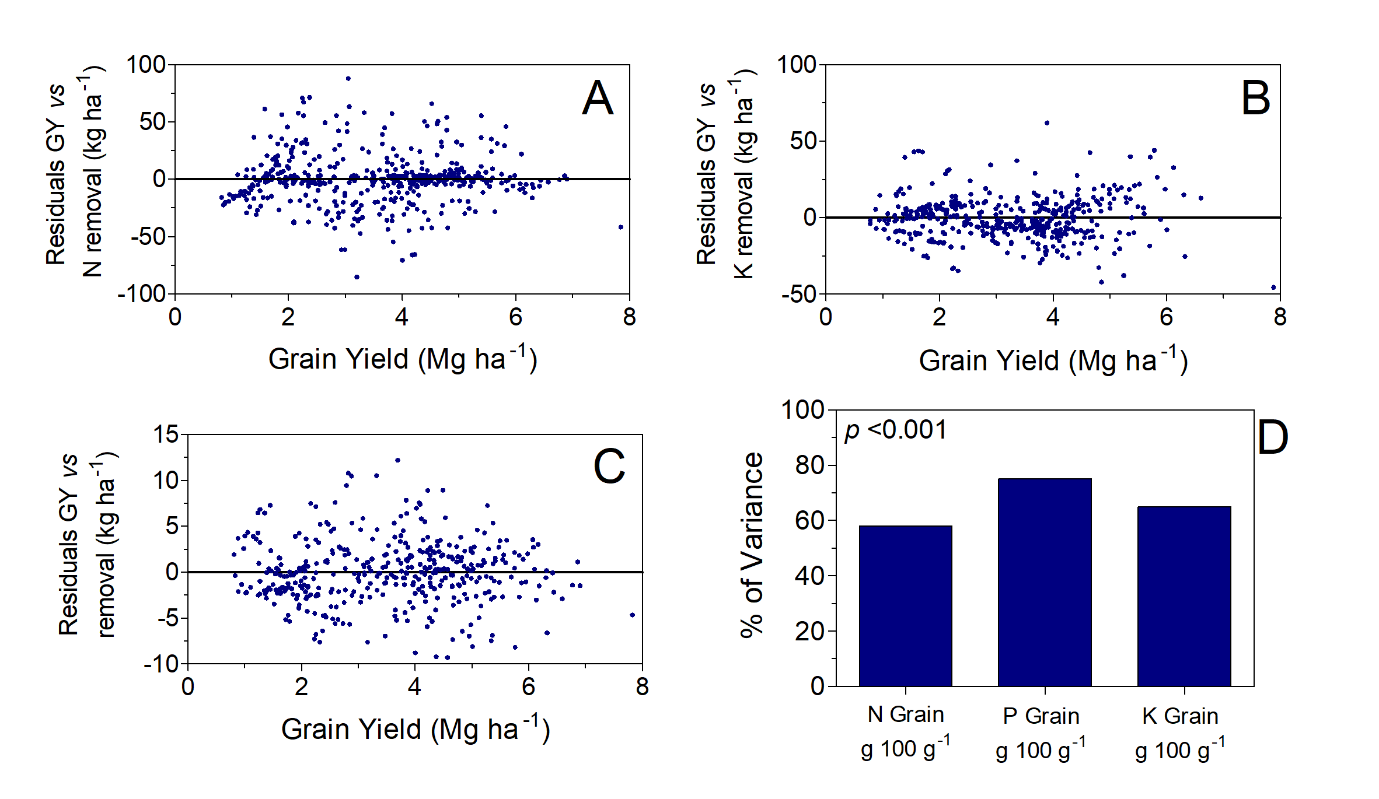


Supplementary figure 1 Scatter plots “A”, “B” and “C” indicated residues of linear function between grain nutrient content and grain yield (Fig. 2A, B and C, respectively) as a function of grain yield values for N, P and K, respectively. Vertical bars plot “D” indicated percentage of variance (R^2^) provided by the linear regression between residuals of Fig. 2A, B and C as a function of their respective grain concentration of N, P and K.


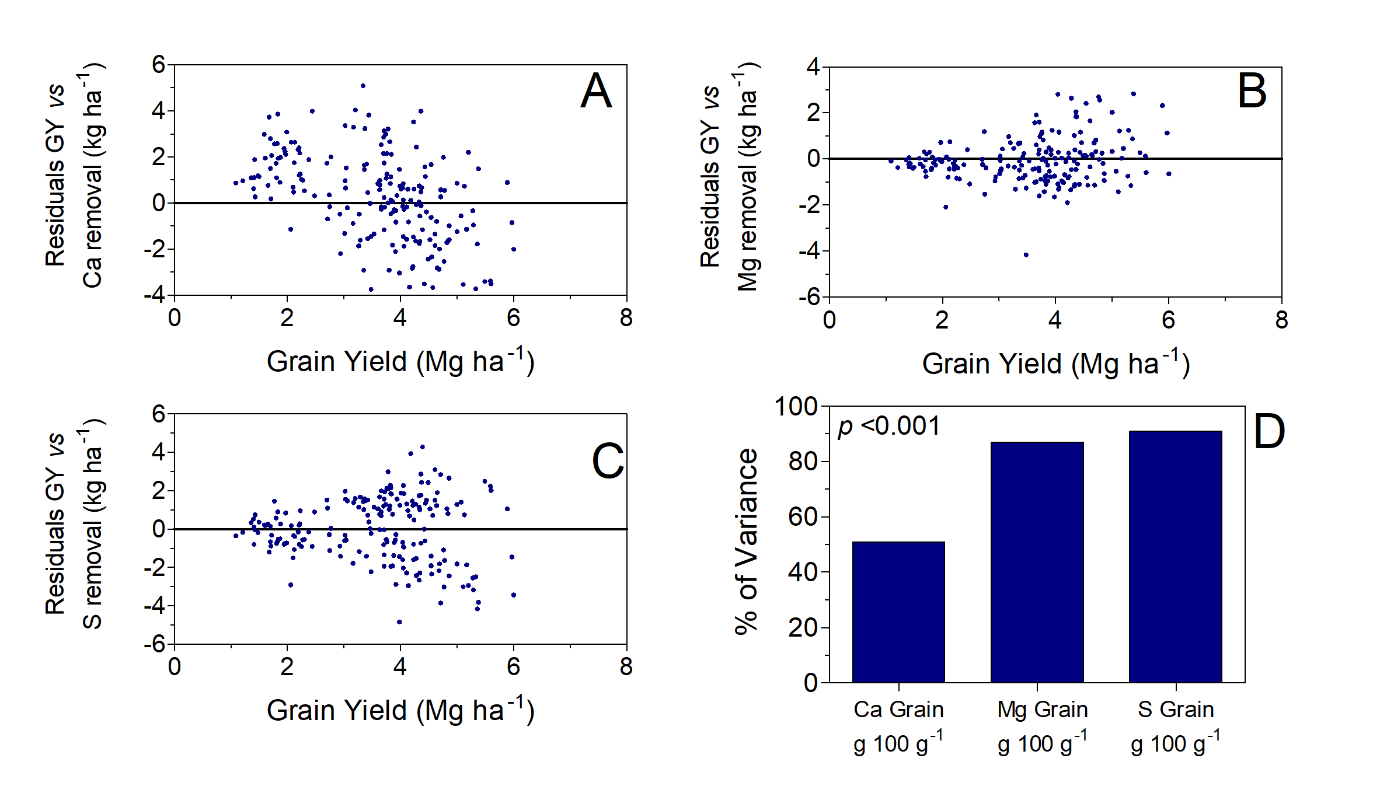


Supplementary figure 2 Scatter plots “A”, “B” and “C” indicated residues of linear function between grain nutrient content and grain yield (Fig. 3A, B and C, respectively) as a function of grain yield values for Ca, Mg and S, respectively. Vertical bars plot “D” indicated percentage of variance (R^2^) provided by the linear regression between residuals of Fig. 3A, B and C as a function of their respective grain concentration of Ca, Mg and S.
